# Benign to Malignant, Malignant to Metastatic: Phyllodes Tumor Biology Transformation

**DOI:** 10.7759/cureus.62487

**Published:** 2024-06-16

**Authors:** Lajpat Rai, Sidra Gulzar, Ali Naqi, Ghina Awais, Sheeraz S Siddiqui

**Affiliations:** 1 Department of General Surgery, Indus Hospital & Health Network, Karachi, PAK; 2 Department of General Surgery, Ysbyty Gwynedd, Bangor, GBR

**Keywords:** surgical management, metastasis, recurrence, malignant transformation, breast neoplasms, phyllodes tumor

## Abstract

Phyllodes tumors (PTs) of the breast are rare fibroepithelial neoplasms, typically characterized by their benign nature. We present a unique case of a 29-year-old Pakistani female who initially presented with a benign PT in her left breast. Despite undergoing multiple surgical resections over the course of a decade, the tumor exhibited a remarkable transformation in biology, progressing from a benign phenotype to malignancy. Subsequent recurrences manifested with increasing aggressiveness, ultimately culminating in distant metastasis to the bones, axillary nodes, chest wall, and abdominal wall. This case underscores the unpredictable nature of PTs and highlights the challenges in managing recurrent cases with malignant transformation. The clinical course described herein emphasizes the importance of vigilant monitoring and individualized treatment strategies in such cases.

## Introduction

Phyllodes tumors (PTs) of the breast are rare fibroepithelial neoplasms, representing approximately 0.5% of all primary breast tumors. While most PTs exhibit a benign clinical course, a subset demonstrates malignant potential, with reported rates ranging from 18% to 25% [1]. These tumors typically present in young to middle-aged females and often mimic the clinical and radiological features of more common fibroadenomas. Histologically, PTs are characterized by increased stromal cellularity, prominent stromal condensation, and leaf-like architecture, distinguishing them from benign fibroadenomas.

Surgical resection with wide margins remains the cornerstone of treatment for PTs, aiming to achieve negative margins and reduce the risk of local recurrence [2]. However, the clinical behavior of PTs can be unpredictable, with recurrences occurring despite apparently adequate initial excision. Furthermore, a subset of PTs may undergo malignant transformation, leading to more aggressive disease manifestations and distant metastasis.

## Case presentation

A 29-year-old Pakistani female initially presented in September 2012 with a palpable left breast mass. Ultrasonography revealed a lesion suggestive of a PT, measuring 6.1 x 3.1 cm, prompting surgical intervention in the form of mastectomy. Microscopic examination of the excised tissue confirmed a diagnosis of a benign PT.

In August 2018, the patient returned with swelling over the left mastectomy scar. A biopsy of the palpable mass revealed benign tissue, leading to close monitoring without immediate intervention.

**Figure 1:**
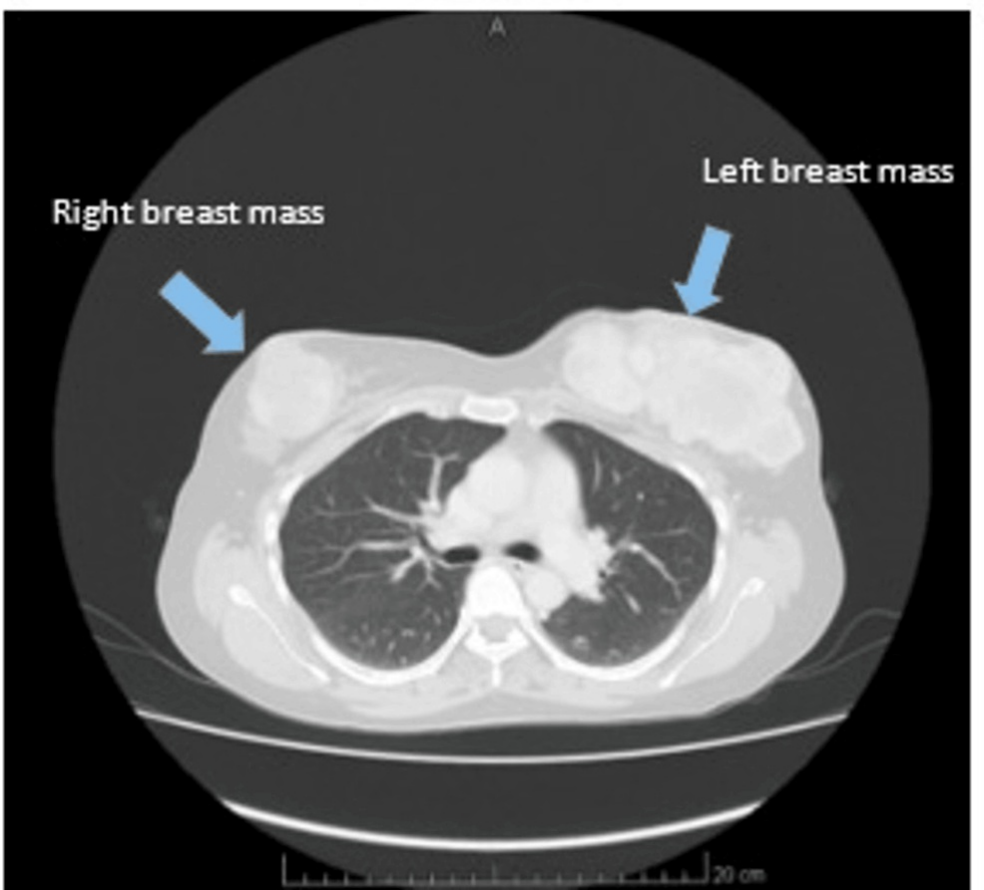
CT imaging (taken October 2019) demonstrated multiple well-defined soft tissue masses with areas of necrosis, with the largest lesion measuring 7.3 x 5.4 cm in the left breast.

The left mastectomy scar swelling progressed, prompting another evaluation in October 2019. Clinical examination revealed bilateral breast swellings, with a new mass in the right breast measuring 3 x 3 cm and a larger lump in the left breast measuring 10 x 10 cm (Figure 1). Excision of the bilateral breast lumps revealed a borderline PT in the right breast and a malignant PT in the left breast, indicating a change in tumor biology.

**Figure 2:**
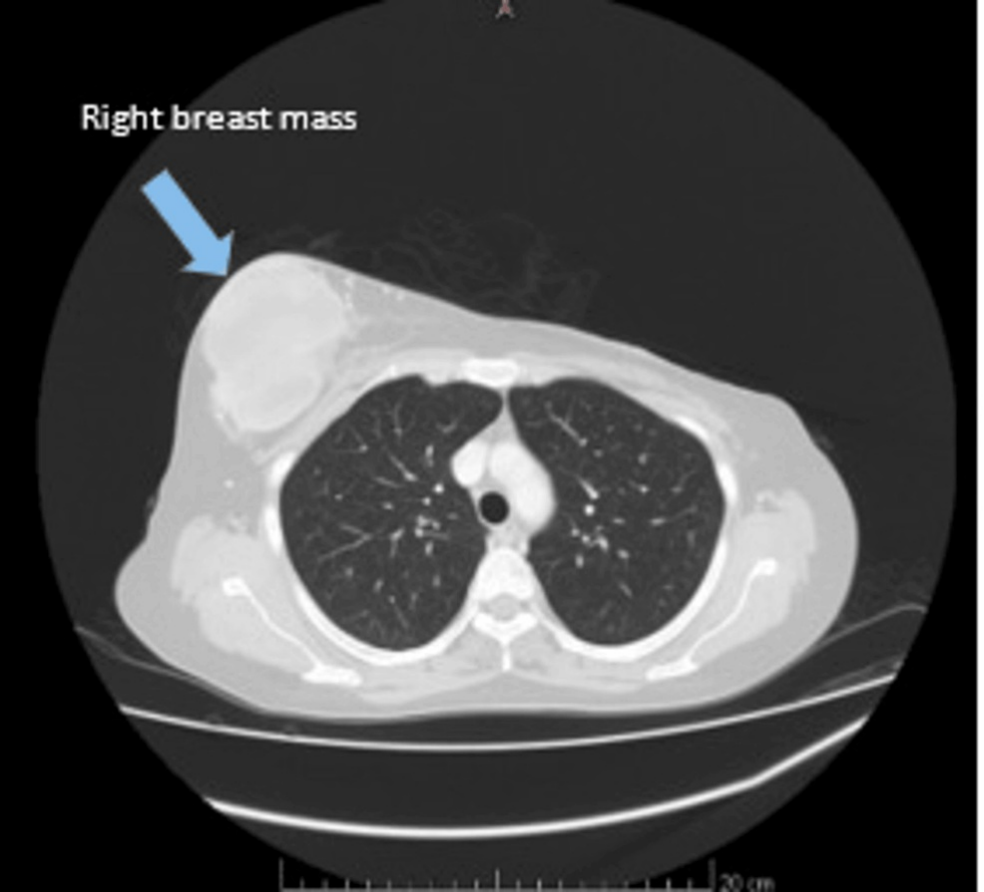
CT imaging (taken July 2020) recurrent swelling in the right breast (blue arrow).

In July 2020, the patient presented with recurrent swelling in the right breast, characterized by a large tumor with overlying skin changes (Figure 2). Subsequent right mastectomy revealed malignant PTs, reflecting a progression in disease aggressiveness.

**Figure 3:**
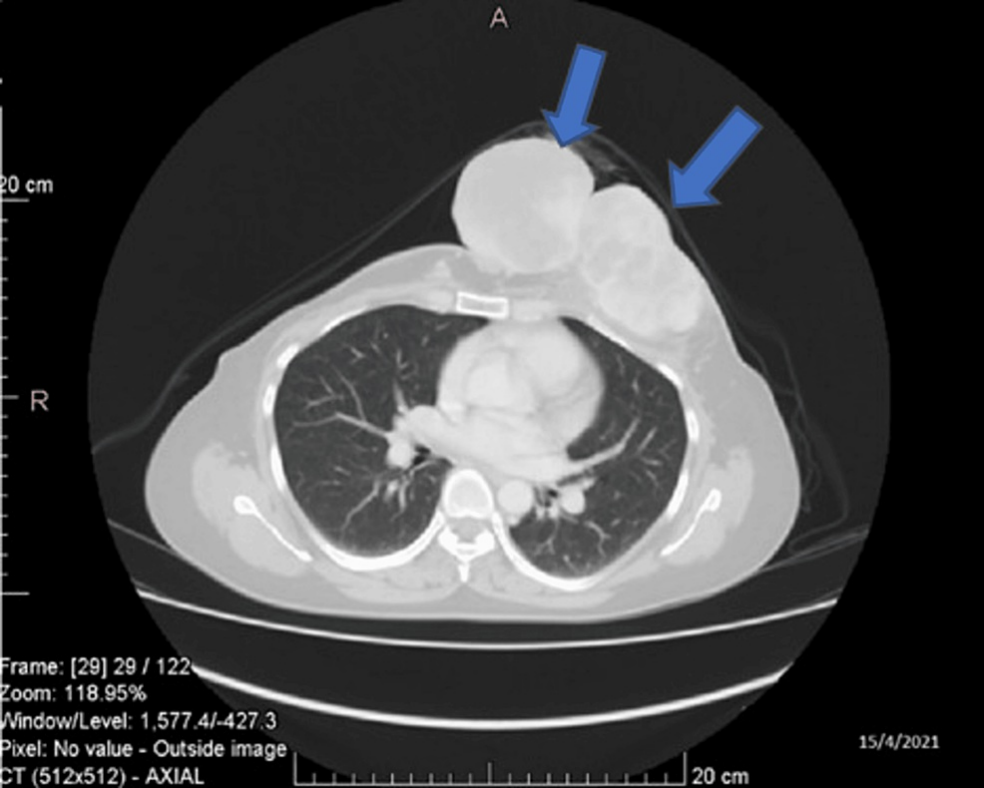
CT imaging (taken April 2021) demonstrated a left anterior chest wall mass (blue arrows).

The patient experienced recurrent disease, culminating in a left anterior chest wall mass in April 2021 (Figure 3). Surgical excision revealed a recurrent PT, with positive margins. Despite attempts, achieving negative margins proved challenging, and the patient declined radiotherapy as an alternative.

**Figure 4:**
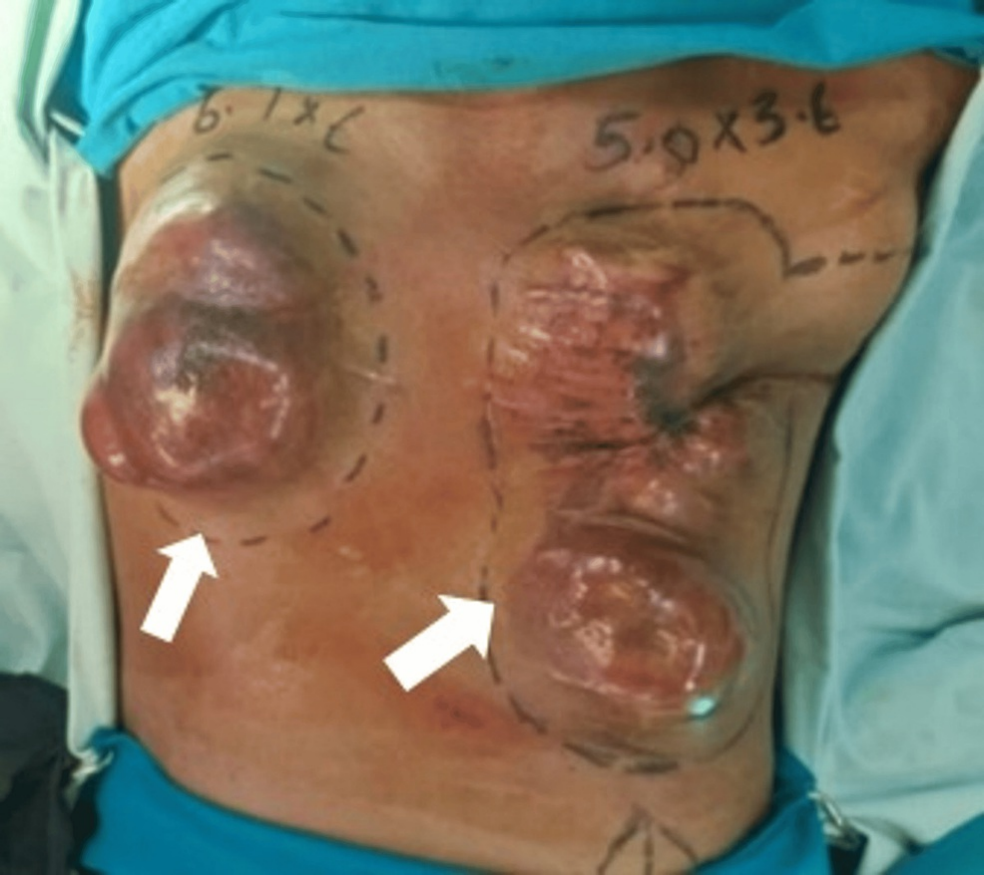
Preoperative tumor burden.

**Figure 5:**
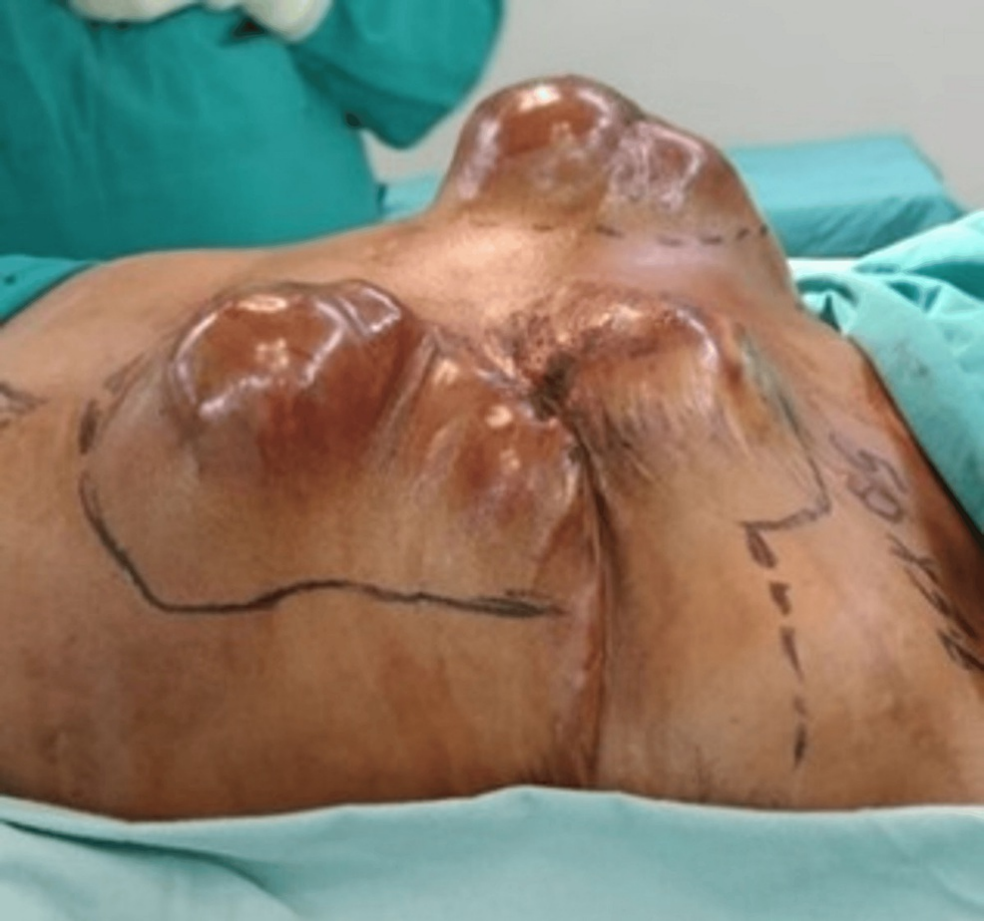
Preoperative tumor burden.

**Figure 6:**
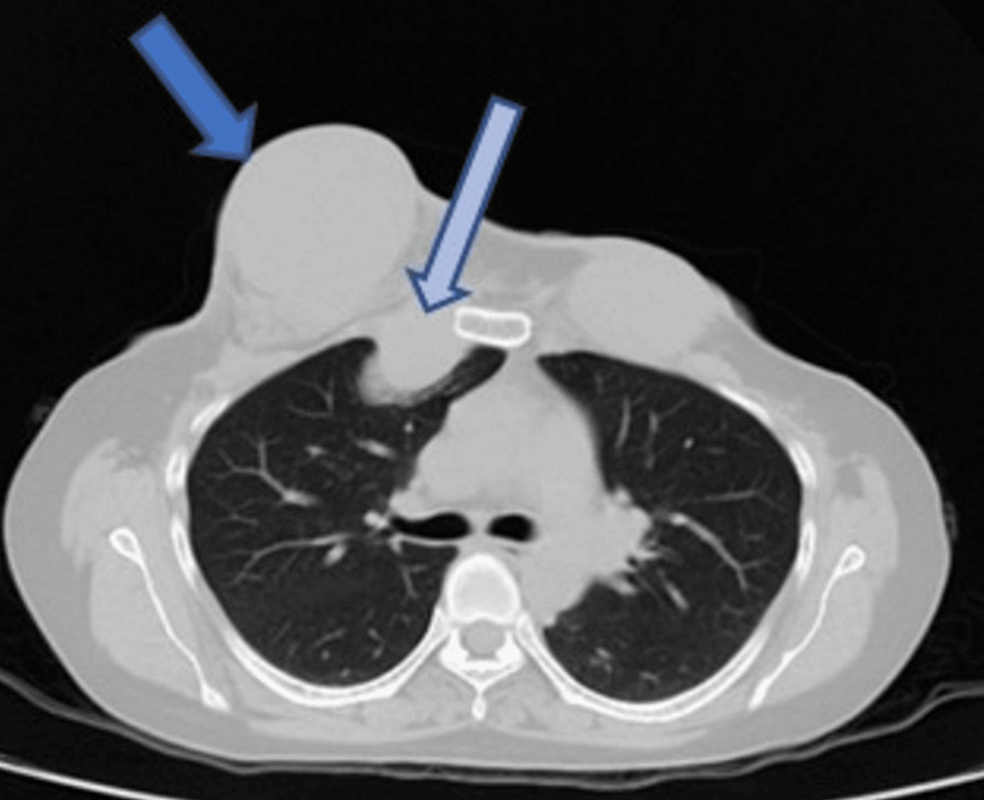
2022 CT scan. The long arrow shows tumor extension in the chest wall, while the shorter arrow indicates the tumor burden on the right side.

**Figure 7:**
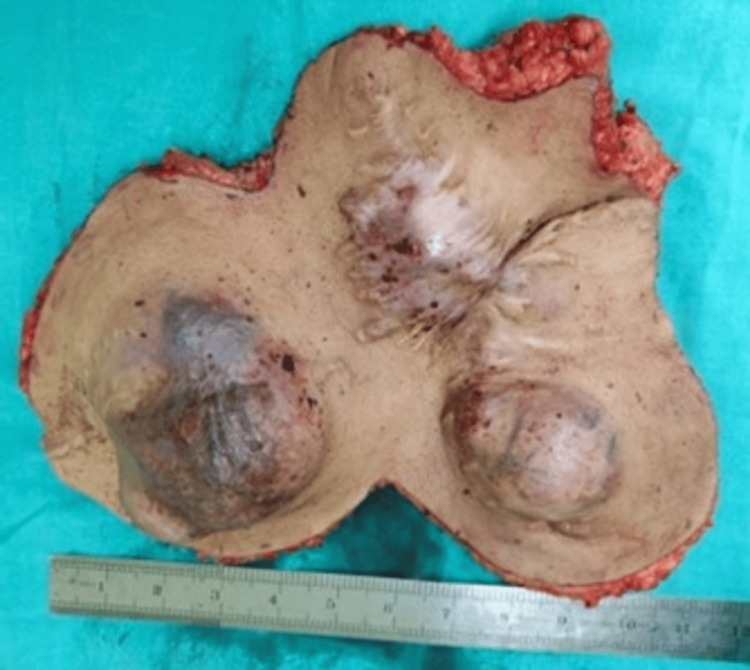
Perioperative tissue specimen.

**Figure 8:**
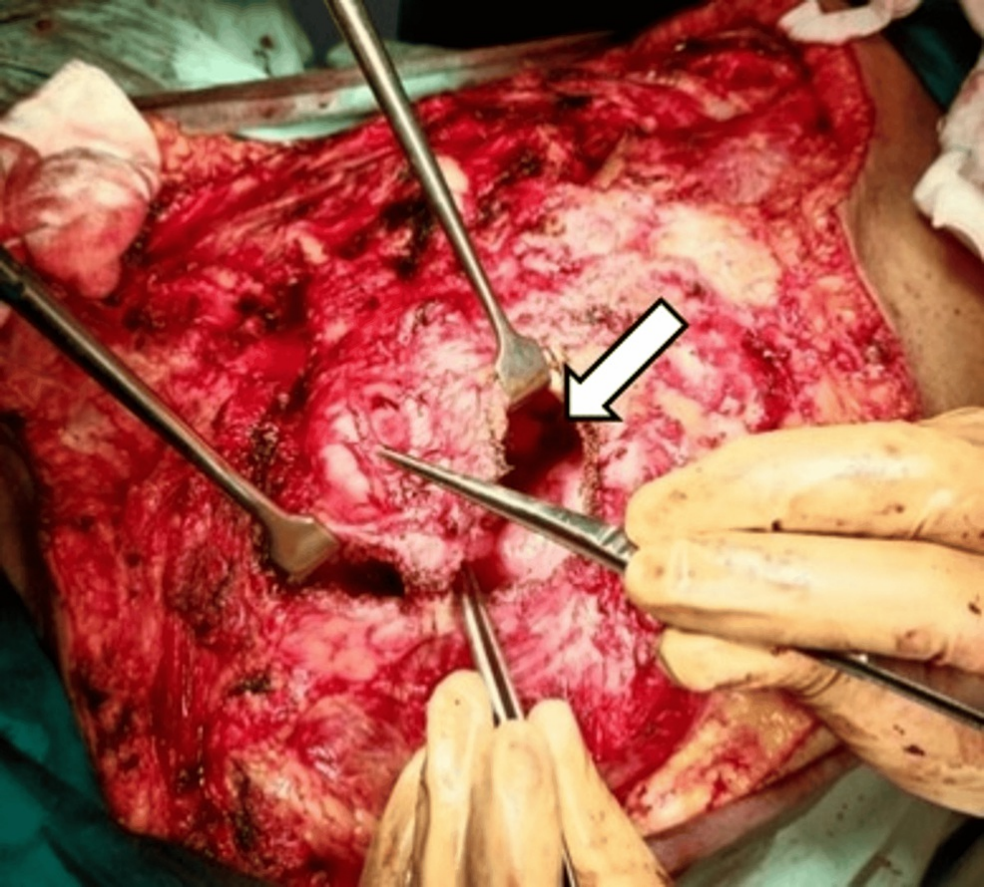
Defect after excision of ribs and lateral edge of sternum in order to get negative margins (shown by the arrows).

**Figure 9:**
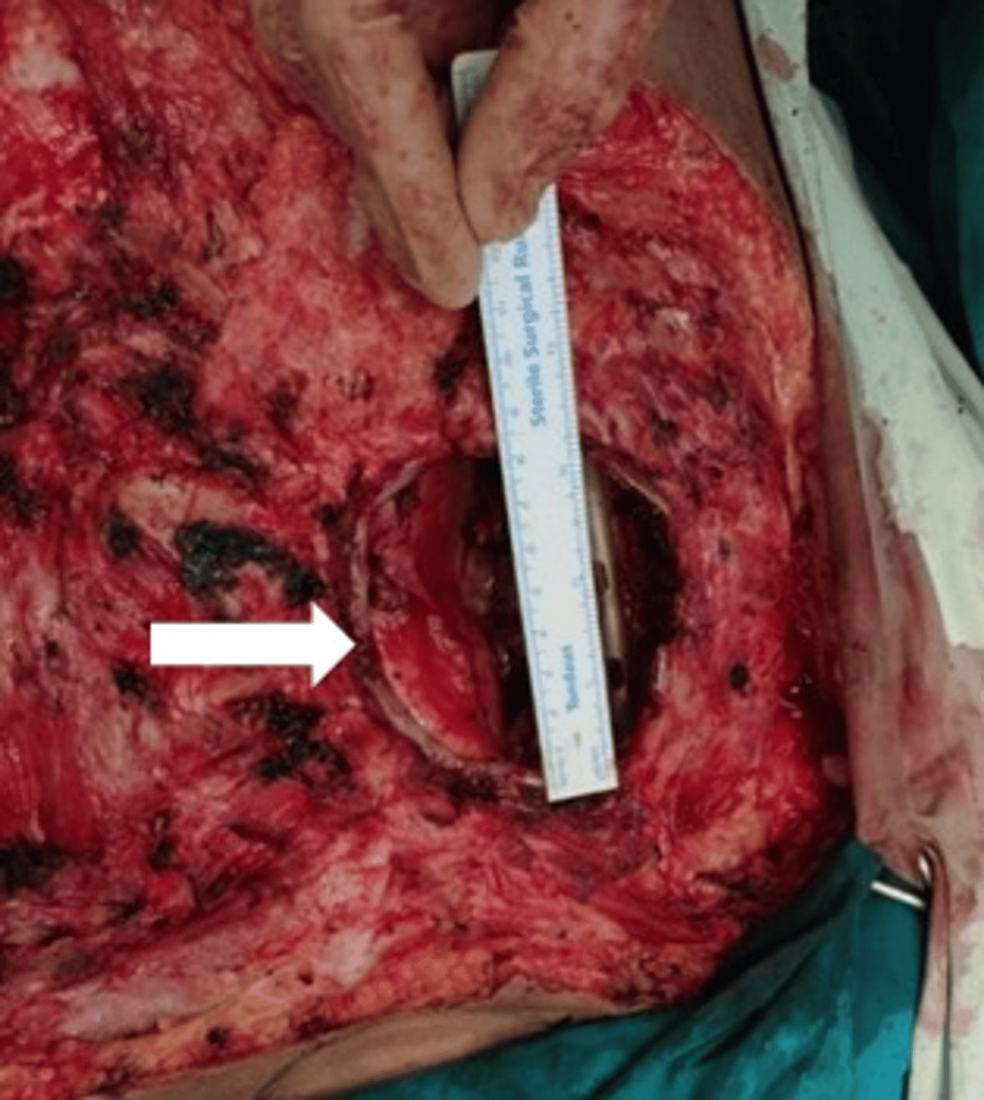
An almost 10 cm defect in the chest wall indicated by the white arrow, which was closed by doing thoracoplasty.

**Figure 10:**
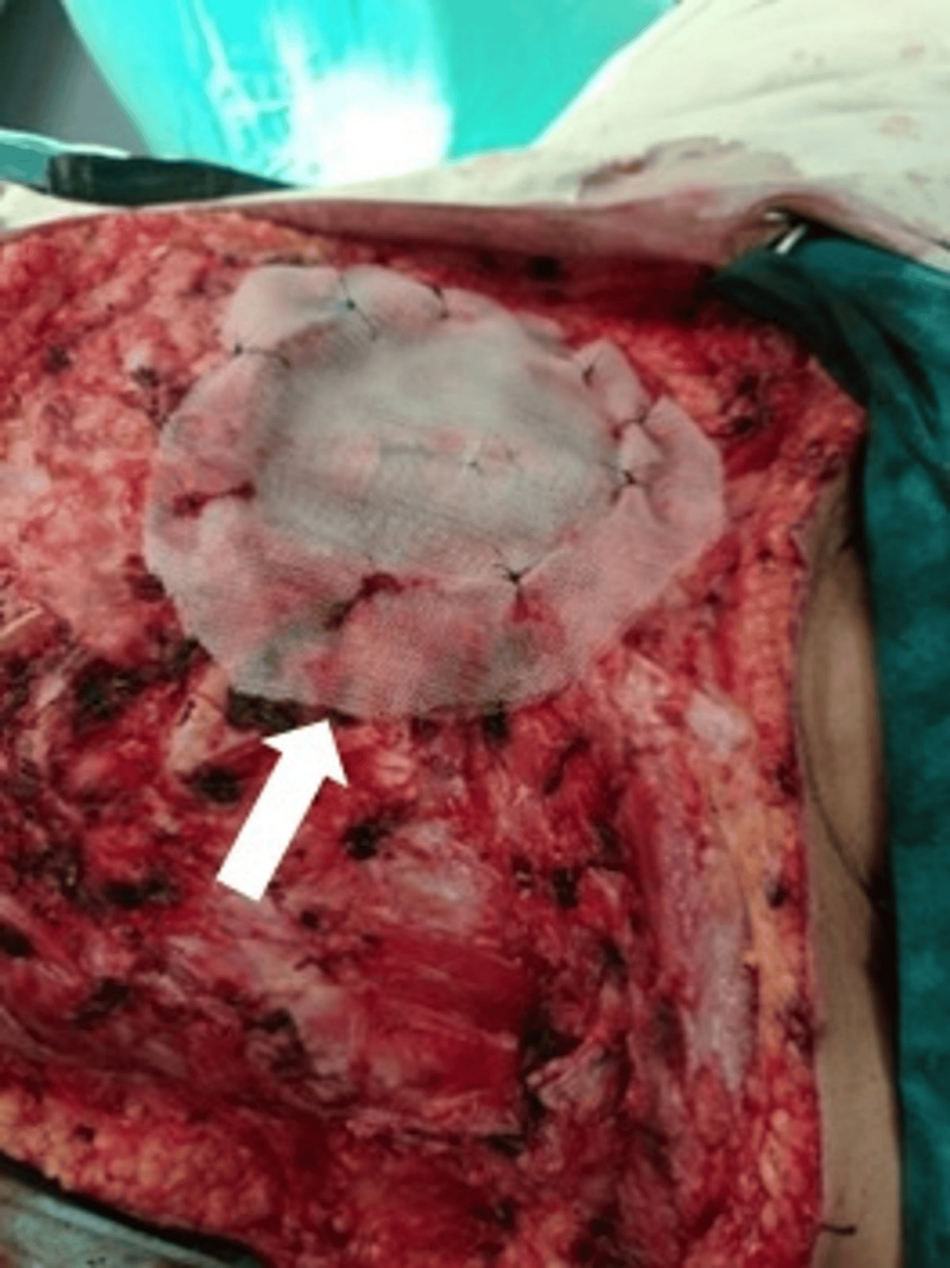
Thoracoplasty with proline mesh.

**Figure 11:**
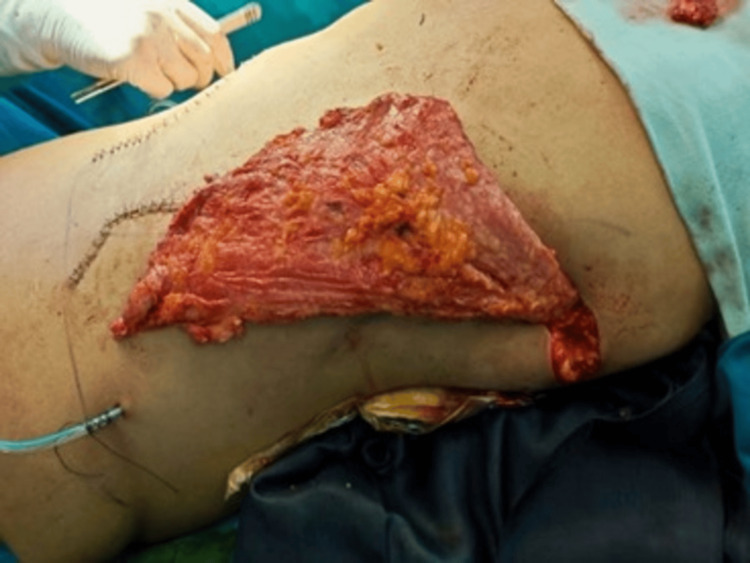
Latissimus dorsi flap.

**Figure 12:**
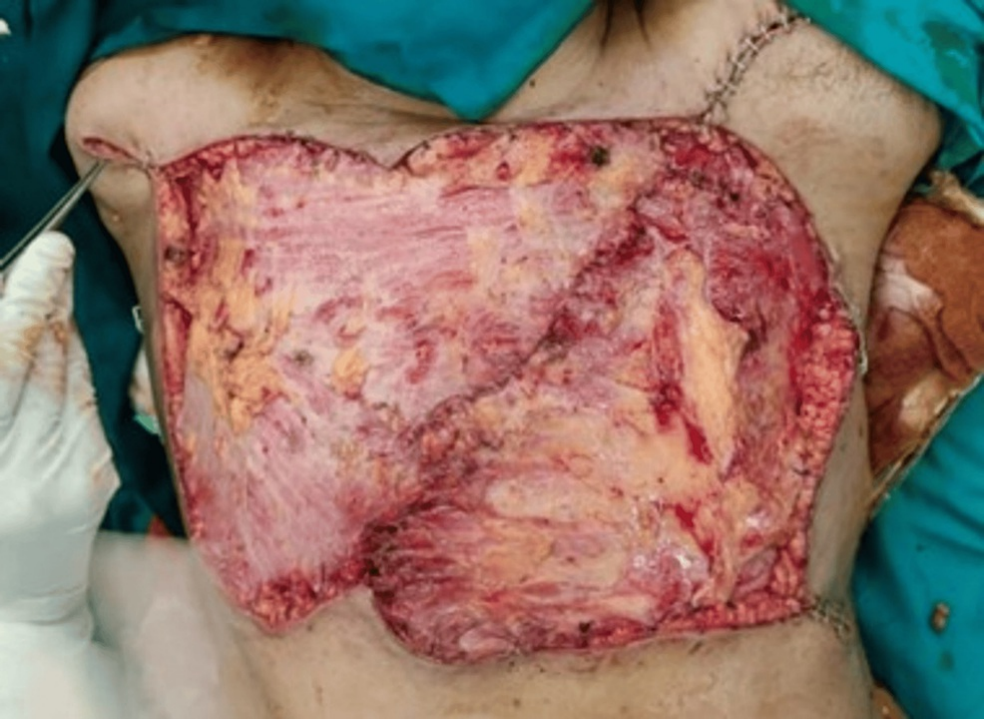
Soft tissue coverage with a bilateral Latissimus dorsi flap.

**Figure 13:**
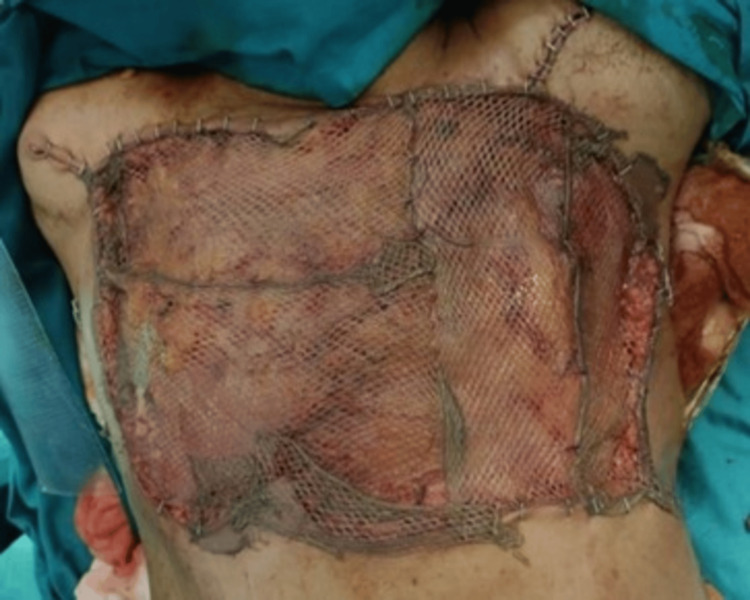
Skin grafting.

**Figure 14:**
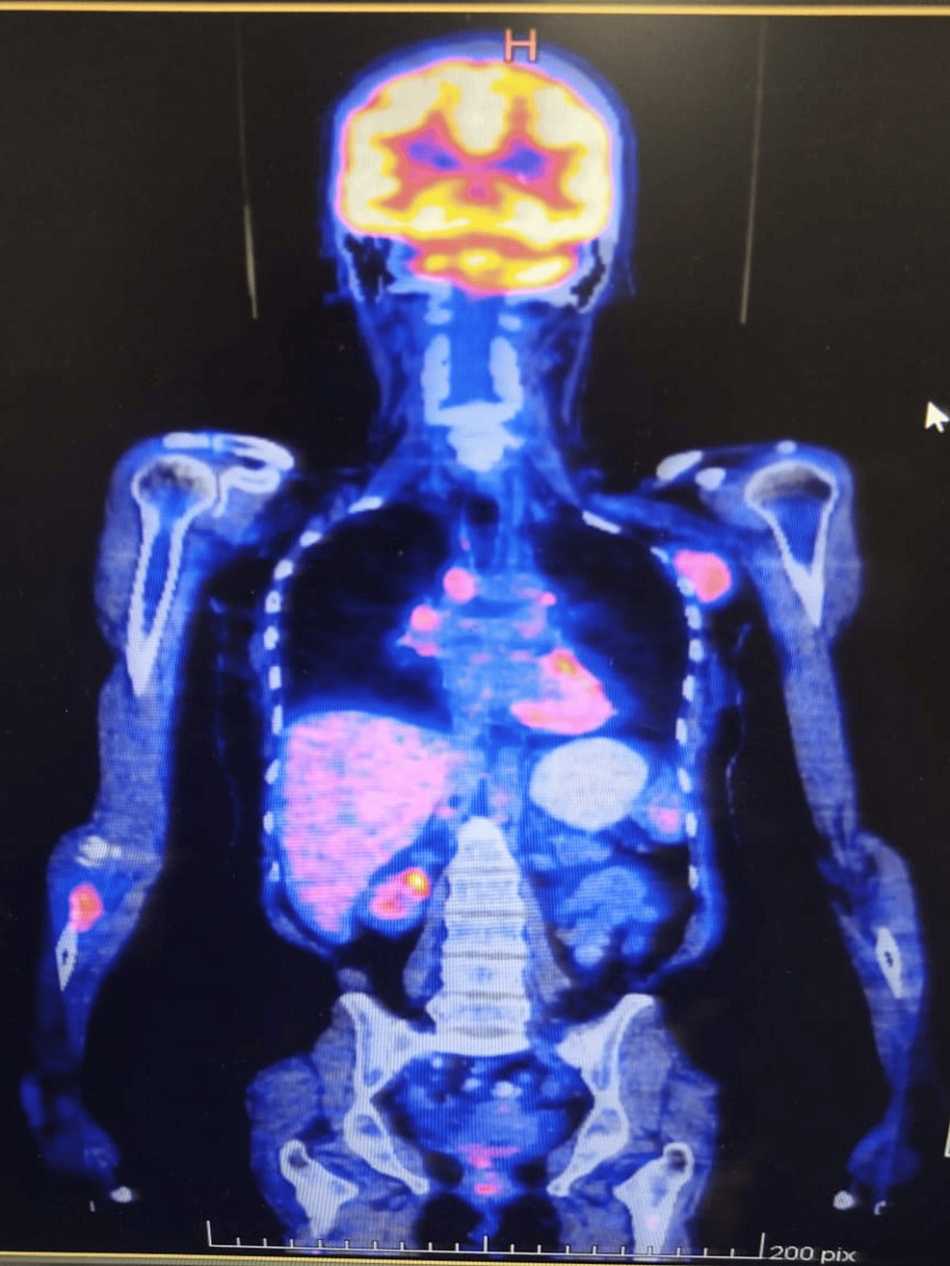
PET scan with the left axilla and right ulna metastasis.

In May 2022, the patient presented with aggressive tumor recurrence and chest wall extension (Figures 4, 5, 6). After thorough discussions, a multidisciplinary team decided on a course of surgery followed by radiotherapy. Coordination between the thoracic sarcoma surgeon and the reconstructive surgeon led to a comprehensive plan. The surgery (August 2022) involved a wide local excision of the tumor and removal of the anterior chest wall to achieve negative margins (Figures 7, 8, 9). Reconstruction of the chest wall was accomplished using thoracoplasty and soft tissue coverage with a latissimus dorsi flap and split-thickness skin grafting (Figures 10, 11, 12, 13). Subsequent follow-up revealed distant metastasis to the bones, chest wall, axilla, and anterior abdominal wall on the PET CT scan in December 2022 (Figure 14). Palliative chemotherapy was not pursued due to poor evidence, and the focus shifted to pain management. Tragically, the patient succumbed to the disease one year after the last surgery.

## Discussion

Previously reported Surveillance, Epidemiology, and End Results (SEER) data estimated the average annual incidence of malignant PTs to be 2.1 cases per million women [3].

There is no specific clinical feature that can differentiate a benign PT from a malignant one. However, a diagnosis of PT is more likely if the tumor is larger than four centimeters or if there is a history of rapid growth. Multifocal PTs are rarely detected in a single breast; they are usually associated with a solitary unilateral mass. Large tumors can invade and ulcerate the skin or extend into the chest wall. A population-based study reported the highest incidence of PT in women aged 45 to 49 years [4].

Ductal elements may be present in locally recurrent PTs in the breast. A post-mastectomy recurrence of a chest wall lesion with ductal elements likely involves focal residual breast tissue. The morphology of recurrent tumors is generally similar to that of the primary PT, but recurrent PTs sometimes differ from the primary tumor and are usually of a higher grade. Most fully malignant PTs are high-grade spindle cell tumors with a fibrosarcomatous appearance, which is the most common appearance in metastatic lesions [4].

PTs are more common in women in the fourth decade of life. They typically present as palpable, painless, rapidly growing breast masses arising from the intralobular stroma, disrupting lobules and ducts. While the epithelial elements are present, only the stromal component is neoplastic. Benign PTs represent 60% to 75% of all cases, characterized by stromal cellularity, minimal stromal atypia, well-defined borders, and no stromal proliferation. These tumors are unlikely to metastasize but have the potential for local recurrence. Malignant PTs, occurring in 10-20% of all PTs, show marked cellular atypia, significant changes in nuclear size and irregular membranes, prominent stromal atypia, infiltrative margins, and stromal proliferation. Borderline PTs represent 15% to 20% of all PTs and often have intermediate features or do not fully meet the criteria for malignancy.

Core biopsy is the preferred method for evaluating breast lesions [1]. Metastasis occurs in malignant and borderline tumors, but metastatic spread of histologically benign tumors has also been noted. Some authors suggest that malignant histology is associated with pain, skin changes, nipple retraction, rapid growth, or a sudden increase in tumor size [2]. The patient in our case experienced painful chest swelling and rapid growth in the tumor size within a matter of months. The skin was shiny and erythematous, suggestive of a compression effect due to rapid growth in the tumor. Malignant PTs can show an unusually aggressive course, with local recurrence and the potential for distant metastasis [3]. Surgical-wide excision with negative margins (≥1 cm) and mastectomy can achieve high local control rates of 80% to 100%. Although PT can be cured by limited surgical excision, all PTs can recur regardless of histology. Chemotherapy, hormonal therapy, and radiotherapy have no proven benefits in preventing PT recurrence and metastasis [4]. Follow-up should align with invasive breast cancer guidelines, with clinical follow-up every three to six months for five years, transitioning to annual exams with mammography thereafter [1].

The presented case highlights the aggressive nature of PTs, with recurrent and bilateral tumors suggesting a possible genetic predisposition. We observed that recurrent tumors exhibit different and more aggressive biology, and achieving negative margins in previously operated sites is difficult. Re-excision to ensure negative margins is crucial to minimize recurrence risk. Such cases should be discussed in tumor board meetings to emphasize the importance of achieving negative margins during surgery. Strict follow-ups are essential for early detection and management of recurrent tumors.

This case underscores the importance of histologically examining all excised breast lesions and performing wide excision of PT. It serves as an example for surgeons dealing with PTs and planning for the ultimate excision of breast and chest wall tumors.

## Conclusions

This case highlights the aggressive nature of PTs in younger females, with a higher risk of malignant transformation and metastasis, likely influenced by genetic factors and margin status. Effective surgical techniques and strict follow-ups are essential to prevent local recurrence, although the optimal margins of excision remain controversial. A PET scan is recommended before extensive surgery to rule out distant metastasis. PTs require histological diagnosis, and a multidisciplinary approach is crucial for treatment planning. Regular early follow-ups are necessary to monitor and detect recurrence in both benign and malignant PTs.
